# Human Pluripotent Stem Cells and Derived Neuroprogenitors Display Differential Degrees of Susceptibility to BH3 Mimetics ABT-263, WEHI-539 and ABT-199

**DOI:** 10.1371/journal.pone.0152607

**Published:** 2016-03-31

**Authors:** Carolina Paola García, Guillermo Agustín Videla Richardson, Nicolás Alexis Dimopoulos, Damián Darío Fernandez Espinosa, Santiago Gabriel Miriuka, Gustavo Emilio Sevlever, Leonardo Romorini, María Elida Scassa

**Affiliations:** Laboratorios de Investigación Aplicada a Neurociencias, LIAN-CONICET, Fundación FLENI, Ruta 9, Km 53, (B1625XAF) Escobar, Buenos Aires, Argentina; University of Colorado, School of Medicine, UNITED STATES

## Abstract

Human embryonic stem cells (hESCs) are hypersensitive to genotoxic stress and display lower survival ability relative to their differentiated progeny. Herein, we attempted to investigate the source of this difference by comparing the DNA damage responses triggered by the topoisomerase I inhibitor camptothecin, in hESCs, human induced pluripotent stem cells (hiPSCs) and hESCs-derived neuroprogenitors (NP). We observed that upon camptothecin exposure pluripotent stem cells underwent apoptosis more swiftly and at a higher rate than differentiated cells. However, the cellular response encompassing ataxia-telangiectasia mutated kinase activation and p53 phosphorylation both on serine 15 as well as on serine 46 resulted very similar among the aforementioned cell types. Importantly, we observed that hESCs and hiPSCs express lower levels of the anti-apoptotic protein Bcl-2 than NP. To assess whether Bcl-2 abundance could account for this differential response we treated cells with ABT-263, WEHI-539 and ABT-199, small molecules that preferentially target the BH3-binding pocket of Bcl-xL and/or Bcl-2 and reduce their ability to sequester pro-apoptotic factors. We found that in the absence of stress stimuli, NP exhibited a higher sensitivity to ABT- 263 and WEHI-539 than hESCs and hiPSCs. Conversely, all tested cell types appeared to be highly resistant to the Bcl-2 specific inhibitor, ABT-199. However, in all cases we determined that ABT-263 or WEHI-539 treatment exacerbated camptothecin-induced apoptosis. Importantly, similar responses were observed after siRNA-mediated down-regulation of Bcl-xL or Bcl-2. Taken together, our results suggest that Bcl-xL contrary to Bcl-2 contributes to ensure cell survival and also functions as a primary suppressor of DNA double-strand brake induced apoptosis both in pluripotent and derived NP cells. The emerging knowledge of the relative dependence of pluripotent and progenitor cells on Bcl-2 and Bcl-xL activities may help to predict cellular responses and potentially manipulate these cells for therapeutic purposes in the near future.

## Introduction

Cells activate survival and/or death signaling pathways under stress conditions. Programmed cell death or apoptosis signaling frequently converges on mitochondria, a process that is controlled by the activities of pro- and anti-apoptotic B-cell lymphoma 2 (Bcl-2) family members [1–3].

Bcl-2 family members can be divided into three main subclasses that are partly defined by the homology shared within four conserved regions. These regions, termed Bcl-2 homology (BH) 1–4 domains, correspond to *α*-helices with similar sequences that determine protein structure and function. In general, the anti-apoptotic members (e.g. Bcl-2, Bcl-xL, Bcl-w and Mcl-1) display sequence homology in all four BH domains, whereas the pro-apoptotic members (e.g. Bax and Bak) have homologous BH1-3 domains [4]. A larger group of pro-apoptotic proteins (e.g. Bid, Bad, Bik, Bim, Bmf, Puma, Noxa and Hrk/DP5) contain only the BH3 domain. The BH3-only proteins are further subdivided into two groups based on function: the “activators” (e.g. Bid, Bim and Puma) and the “sensitizers” (e.g. Bad, Bik and Noxa) [5, 6].

A key initial step in apoptotic processes is the activation of Bax or Bak by the formation of dimers through the binding of the BH3 domain of one Bax or Bak molecule to a hydrophobic groove of a second Bax or Bak [7]. Bax and Bak oligomers participate in the formation of pores in the outer mitochondrial membrane and promote the release of mitochondrial factors that mediate caspases activation and the hallmark features of apoptosis. This oligomerization can be induced by numerous signals, such as p53-induced BH3-only proteins or by p53 itself [8, 9].

Pro-survival Bcl-2 family members counteract apoptosis by sequestering BH3 domains of both multidomain pro-apoptotic proteins and BH3-only proteins. Activator BH3-only proteins bind to and inhibit anti-apoptotic members (e.g. Bcl-2, Bcl-xL, and Mcl-1), and also directly interact with Bax and Bak causing their oligomerization at the mitochondria [1]. Instead, sensitizer BH3-only proteins can only bind to anti-apoptotic factors allowing the release of activator and pro-apoptotic proteins to drive mitochondrial outer membrane permeabilization [5, 10, 11].

When BH3-only proteins are activated in response to stress signals, the BH3-binding pockets of pro-survival proteins become saturated and Bax or Bak activation is then able to proceed unchecked [12]. Small molecules that mimic BH3 motifs are expected to induce apoptosis by binding to the hydrophobic groove of anti-apoptotic Bcl-2 members and displacing bound activators BH3-only proteins.

Human embryonic stem cells (hESCs) are derived from the inner cell mass of pre-implantation blastocysts, whereas induced pluripotent stem cells (hiPSCs) are generated from the reprogramming of somatic cells back to a pluripotent state [13, 14]. Both cell types have the potential to generate almost any cell type in the human adult organism and for this reason it is assumed that the maintenance of their genome integrity is critical.

Pluripotent cells are exceptionally sensitive to exogenous stressors, such as DNA damaging agents, when compared to their differentiated counterparts and quickly undergo apoptosis rather than attempting repair of a compromised genome [15]. Currently, the mechanisms behind this apoptosis-prone state are not well understood. Recently, Liu and coworkers [16] reported that the susceptibility of hESCs to undergo apoptosis may be related to their higher state of mitochondrial priming, a lowered cell intrinsic threshold for initiating apoptosis, based on the balance of pro- and anti-apoptotic proteins.

Neural progenitors (NP) derived from hESCs have been developed as a model to study neurodevelopment and neurotoxicity. These NP are capable of self-renewal and differentiation into neural lineage cells. Understanding the basic biology of NP is necessary for generating functional differentiated cells that may be used both, as an *in vitro* model and ultimately to replace dysfunctional or degenerating neurons.

Programmed cell death, involving Bcl-2 family proteins, is an essential mechanism employed by the developing nervous system to remove excess or damaged neurons [17]. However, programmed cell death also becomes aberrantly activated during various neurodegenerative diseases and because of that, remains an important therapeutic target for combating these type of disorders [18]. Thus, the study of NP vulnerability to deleterious DNA damage including DNA double-strand breaks (DSBs) that could result either from naturally occurring metabolic products or from the effect of exogenous stressors results relevant [19].

Herein, in an effort to learn more about how hESCs, hiPSCs and hESCs undergoing neural differentiation protect their genomic integrity against potentially lethal DSBs we compared their response against the topoisomerase I inhibitor, camptothecin (CPT) [20]. We found that the DNA damage response, involving mainly ataxia telangiectasia mutated (ATM) signaling and p53 phosphorylation at serine 15 and 46, was similar in both pluripotent cell types and immature differentiated progeny (NP). We determined that CPT induces caspase-9 and -3 activation, poly (ADP-ribose) polymerase (PARP) cleavage and apoptotic features in pluripotent stem cells and in hESCs-derived NP, although to different degrees and with different kinetics. Moreover, we found that specific inhibition of mitochondrial p53 translocation by Pifithrin-μ (PFT-μ) reduces the apoptotic response triggered by CPT in hiPSCs but not in NP, underlining the significance of p53’s mitochondrial program in pluripotent stem cells apoptosis regulation.

To gain insight into the mechanisms that control hESCs, hiPSCs and hESCs-derived NP fate decisions in response to DSBs, we attenuated their anti-apoptotic activities by using ABT-263, WEHI-539 and ABT-199, small molecules that mimic BH3 motifs. ABT-263 preferentially targets the BH3-binding pockets of Bcl-2 and Bcl-xL while WEHI-539 solely targets Bcl-xl and ABT-199 selectively inhibits Bcl-2 [21–23]. Using these agents we studied the contribution of Bcl-xL and/or Bcl-2 inhibition in stem and progenitor cells survival. We also determined that ABT-263 or WEHI-539 treatment exacerbates apoptosis triggered by CPT. This study envisions a model where Bcl-xL regulates cell survival and operates as a primary suppressor of DSBs-induced cell death in the tested cell types.

## Materials and Methods

This study is in compliance with the October 2013 Helsinki Declaration and it has been approved by the Biomedical Research Ethics Committee “Comité de Ética en Investigaciones Biomédicas de la Fundación para la Lucha contra Enfermedades Neurológicas de la Infancia (FLENI),” and written informed consent was received from each patient whose tissue was used for this project.

### Cell culture

The hESC line, WA09 (H9), was purchased from WiCell Research Institute (WI) at low passages (p15 to p20) [13]. The hiPSC line FN2.1 was generated from human foreskin fibroblasts. Cells were reprogrammed with STEMCCA lentiviruses infection as previously described [24, 25].

hESC and hiPSC lines were maintained on an inactivated mouse embryonic fibroblast (iMEF) feeder layer in medium comprised of Dulbecco's Modified Eagle's Medium/Ham's F12 (DMEM/F12) supplemented with 20% knockout serum replacement (KSR), 2 mM non-essential amino acids, 2 mM L-glutamine, 100 U/ml penicillin, 50 μg/ml streptomycin, 0.1 mM β-mercaptoethanol and 4 ng/ml of basic fibroblast growth factor (bFGF). All these reagents were obtained from Invitrogen (Carlsbad, CA, USA). Pluripotent cells were transferred with 1 mg/ml collagenase IV (Invitrogen, CA, USA) into feeder-free diluted (1/40) Matrigel^™^ (BD Bioscience, San Jose, CA, USA) coated dishes containing iMEF conditioned medium. For conditioning medium, 3×10^6^ inactivated MEFs were incubated for 24 h with 25 ml of DMEM/F12 medium supplemented with 5% KSR and 2 ng/ml of bFGF (in addition to the other aforementioned supplements) and stored at -20°C. After thawing, fresh aliquots of KSR and bFGF were added to the medium to render a final concentration of 20% and 4 ng/ml, respectively.

Human foreskin fibroblasts (HF) were prepared as primary cultures from freshly obtained human foreskins as soon as possible after surgery. Written informed consent was obtained from patients according to guidelines established by the Ethics Committee of the Foundation for the Fight against Pediatric Neurological Disease (FLENI). Briefly, after fat and loose fascia removal, surgical discard tissue was trimmed into strips (approximately 0.5 cm × 1.5 cm) using a sterile scalpel. The cut tissue was subjected to an overnight digestion with dispase and then followed by careful removal of the epidermis. The remaining dermis was placed in high glucose DMEM, 10% FBS (vol/vol), plated onto tissue culture plates and incubated in a 37°C, 5% CO_2_, 90% humidity incubator. Within 7–10 days outgrowths of fibroblasts appeared. The isolated fibroblasts were then expanded, frozen and stored as described elsewhere.

### Generation of neural progenitors

Embryoid bodies (EBs) were generated by cutting H9 colonies into small pieces with a needle, detaching them from the feeder layer and culturing them in an ultralow adhesion culture dishes in DMEM/F12 medium supplemented with 20% KSR, 2 mM non-essential amino acids, 2 mM L-glutamine, 100 U/ml penicillin, 50 μg/ml streptomycin, 0.1 mM β-mercaptoethanol for 4 days. Medium was then replaced with Neural induction medium (DMEM/F12 medium supplemented with N-2 supplement, 2 mM non-essential amino acids and 1 μg/ml heparin). To induce neural rosette formation, 6-day-old EBs were plated on 20 μg/ml laminin-coated (Sigma Aldrich, MO, USA) dishes and cultured in Neural induction medium for 15 days. During the culture of EBs on the laminin-coated surfaces, neural rosettes were observed and manually removed from the surrounding flat cells. Next, the rosettes were dissected into small pieces using a sterile pulled glass pipette under a stereomicroscope and plated on laminin-coated dishes and were cultured in a Neural proliferation medium, which consisted of Neurobasal medium supplemented with B27, N-2, 2 mM L-glutamine, 2mM non-essential amino acids, 50 U/ml penicillin/streptomycin, 20 ng/ml bFGF, 20 ng/ml epidermal growth factor (all purchased from Invitrogen, CA, USA), 20μg/ml bovine pancreas insulin and 75 μg/ml low-endotoxin bovine serum albumin (Sigma, MO, USA). After the initial differentiation lasting 21 days, NP were dissociated using accutase (Invitrogen, CA, USA) for 5 min, centrifuged at 300 x g for 5 min, resuspended with Neural proliferation medium and plated on 10 μg/ml laminin-coated dishes for further expansion and cryopreservation.

For neuronal differentiation, NP were suspended in Neurobasal medium supplemented with B27, N-2, 2 mM L-glutamine, 2mM non-essential amino acids, 50 U/ml penicillin/streptomycin (all from Invitrogen, CA, USA), 20μg/ml bovine pancreas insulin and 75 μg/ml low-endotoxin bovine serum albumin (Sigma, MO, USA) as floating aggregates for three days. Then medium was replaced by Neural induction medium (DMEM/F12 medium supplemented with N-2, 2 mM non-essential amino acids and 1 μg/ml heparin) and cellular aggregates were expanded for 10 days. Medium was changed every 2 days. After neural expansion, the aggregates (neurospheres) were allowed to attach onto 10 μg/ml laminin-coated plates in Neural differentiation medium (Neurobasal medium supplemented with B27, N-2, 10 ng/ml BDNF, 10 ng/ml GDNF, 200 μg/ml ascorbic acid, 0.1 μM cAMP and 20 μg/ml laminin for 10 days. Medium was changed every 2 days.

### Inhibitors

(R)-4-(4-((4'-chloro-4,4-dimethyl-3,4,5,6-tetrahydro-[1,1'-biphenyl]-2-yl)methyl)piperazin-1-yl)-N-((4-((4-morpholino-1-(phenylthio)butan-2-yl)amino)-3 ((trifluoromethyl) sulfonyl) phenyl) sulfonyl) benzamide (ABT-263); 4-[4-[[2-(4-chlorophenyl)-4,4-dimethylcyclohexen-1-yl]methyl]piperazin-1-yl]-N-[3-nitro-4-(oxan-4 ylmethylamino) phenyl] sulfonyl-2-(1H-pyrrolo[2,3-b]pyridin-5-yloxy) benzamide (ABT-199) (Santa Cruz Biotechnology, Santa Cruz, CA); 5-[3-[4-(aminomethyl)phenoxy]propyl]-2-[(8E)-8-(1,3-benzothiazol-2-ylhydrazinylidene)-6,7-dihydro-5H-naphthalen-2-yl]-1,3-thiazole-4-carboxylic acid (WEHI-539) (a generous gift from Genentech USA Inc, San Francisco, CA, USA) and PFT-μ (Sigma, St. Louis, MO, USA) were dissolved in DMSO and stored at -20°C. Inhibitors were added to cell cultures such that the final DMSO concentrations were kept constant at 0.25% (v/v).

### Cell transfection and RNA Interference

Cells were transfected with the corresponding small interfering RNA (siRNA) using Lipofectamine^™^ 2000 lipid reagent (Invitrogen CA, USA) as per the manufacturer's instructions. Briefly, 3x10^5^ cells/well (6-well plate) were transfected with Silencer^®^ Select Negative Control #2 (Ambion^™^, cat#4390846), Silencer^®^ Select Validated Bcl-xL siRNA (Ambion^™^, siRNA ID: 120717) or Silencer^®^ pre-designed Bcl-2 siRNA (Ambion^™^, siRNA ID: 214532) (Invitrogen, CA, USA). The concentration of siRNA used for cell transfection (50 nM) was selected based on dose-response studies. Forty eight hours after transfection cell viability was determined.

### Immunostaining and fluorescence microscopy

hiPSCs and hESCs-derived NP were analyzed for in situ immunofluorescence. Briefly, cells were rinsed with ice-cold PBS and fixed in PBSA (PBS with 0.1% bovine serum albumin) with 4% formaldehyde for 45 min. After two washes with PBS, cells were permeabilized with 0.1% Triton X-100 in PBSA with 10% normal goat serum for 30 min, washed twice and stained with the corresponding primary antibodies. Fluorescent secondary antibodies were used to localize the antigen/primary antibody complexes. The cells were counterstained with DAPI and examined under a Nikon Eclipse TE2000-S inverted microscope equipped with a 20X E-Plan objective and a super high-pressure mercury lamp. The images were acquired with a Nikon DXN1200F digital camera, which was controlled by the EclipseNet software (version 1.20.0 build 61). The following primary antibodies were used: α-phosphoATM (ab81292), α-γH2AX (ab2893), α-p53 (ab1101) (Abcam Inc., Cambridge, MA, USA), α-phospho-p53^Ser15^ (cat.9284) (Cell Signaling Technology, Beverly, MA, USA); α-MAP2 (M1406), α-MAP5 (M4528) (Sigma, St. Louis, MO, USA); α-nestin (AB5922) (Millipore, Temecula, CA, USA) and α-doublecortin (E-6) (sc-271390) Santa Cruz, CA, USA), α-Tuj1 (MMS-435P) (Covance, Princeton, NJ, USA)

### Flow cytometric analysis

Single-cell suspensions of NP were obtained by treatment with acutasse (Invitrogen, CA, USA) (37°C for 5–10 min). Cells (1x10^6^) were incubated for 30 min at 4°C with monoclonal CD133/1 (AC133)-PE conjugate antibody (1:40, Miltenyi Biotec, Auburn, CA, USA) and washed with 2 ml of PBS. Cells were then centrifuged at 200 x *g* for 5 min, resuspended in 0.5 ml PBS and analyzed by flow cytometry. Data was acquired on a FACSAria II flow cytometer from Becton Dickinson (BD Biosciences, San Jose, USA) using WinMDI 2.9 software. Background fluorescence was estimated by substituting the specific primary antibody with specific isotype controls.

### Flow cytometric analysis of cell viability using Propidium Iodide (PI)

PI is a membrane-impermeant chromatin dye that is excluded from cells with intact membranes, whereas cells with damaged plasma membranes emit red fluorescence as the dye intercalates with DNA. Twenty hours after ABT-263, WEHI-539 or ABT-199 addition, single-cell suspensions were obtained by treatment with acutasse (37°C for 5–10 min). Cells were then centrifuged at 200 x *g* for 5 min and resuspended up to 10^6^ cells/ml in FACS Buffer (2.5 mM CaCl_2_, 140 mM NaCl and 10 mM HEPES, pH 7.4). Next, 100 μl of cellular suspension were incubated with 5 μl of PI (50 μg/ml) in PBS for 5 min in the dark. Finally, 400 μl of FACS Buffer were added to each tube and cells were immediately analyzed by flow cytometry. Results were expressed as the percentage of cells that displayed PI fluorescence (non-viable) to the total number of cells processed. Fluorescence intensity was determined by flow cytometry on a FACScan equipped with a 488-nm argon laser (Becton Dickinson).

### Flow Cytometric Analysis of Cell Cycle Distribution

For DNA content analysis, cells were fixed in 70% ethanol, rehydrated in PBS, and treated for 30 min with RNase A (1 mg/ml) and for 5 min with PI (1 mg/ml). Fluorescence intensity was determined by flow cytometry on a FACScan equipped with a 488-nm argon laser (Becton Dickinson). Data acquisition was performed with the BD CellQuest software, and the percentages of G1, S, and G2/M-phase cells were calculated with the MODFIT-LT software program (Verity Software House, Topsham, ME, USA).

### Reverse transcription polymerase chain reaction

Total RNA was extracted using TRIzol reagent (Invitrogen, Carlsbad, CA, USA) according to manufacturer's instructions. cDNA was synthesized using MMLV reverse transcriptase (Promega, Madison, WI, USA) from 500 ng of total RNA. The cDNA samples were diluted fivefold. Quantitative PCR studies were carried out using SYBR^®^ Green-ER^™^ qPCR SuperMix UDG (Invitrogen, Carlsbad, CA, USA). Primers used were the following: Bcl-2, forward 5'-TATAACTGGAGAGTGCTGAAG-3', reverse 5'-ACTTGATTCTGGTGTTTCCC-3'; Bcl-xL, forward 5'-TGCGTGGAAAGCGTAGACAAG-3', reverse 5'-GTGGGAGGGTAGAGTGGATGG-3'; Bax, forward 5'-GACGGCAACTTCAACTGG-3', reverse 5'-GTGAGGAGGCTTGAGGAG-3'; Bcl-w, forward 5'-TGGATGGTGGCCTACCTG-3', reverse 5'-CGTCCCCGTATAGAGCTGTG-3'; Mcl-1, forward 5'-GGGCAGGATTGTGACTCTCATT-3', reverse 5'-GATGCAGCTTTCTTGGTTTATGG-3'; Puma, forward 5'-GACCTCAACGCACAGTACGAG-3', reverse 5'-AGGAGTCCCATGATGAGATTGT-3' and GAPDH, forward 5'-ACAGCCTCAAGATCATCAG-3', reverse 5'-GAGTCCTTCCACGATACC-3'. All samples were analyzed using an ABI PRISM 7500 Sequence Detector System (Applied Biosystems, Foster City, CA, USA) and were normalized to GAPDH gene expression.

### Western Blotting

Cells were lysed in ice-cold RIPA buffer supplemented with a protease and phosphatase inhibitor mixture, and protein concentration was determined using Bicinchoninic Acid Protein Assay (Pierce^™^, Rockford, IL, USA). Equal amounts of protein were run on 12% polyacrylamide gel electrophoresis, and transferred to PVDF-FL membrane (Millipore, Billerica, MA, USA). The membrane was blocked for 1 h in Odyssey blocking buffer (LI-COR Biosciences, Lincoln, NE, USA) containing 0.1% Tween 20 and then incubated overnight at 4°C in a solution containing Odyssey blocking buffer, 0.05% Tween 20 and the corresponding primary antibodies. The membrane was washed 4 *×* 5 min with Tris-buffered saline (TBS), 20 mM Tris-HCl, pH 7.5, 500 mM NaCl) containing 0.1% Tween 20 (TTBS), then incubated for 1 h in a solution containing Odyssey blocking buffer, 0.2% Tween 20, and IR-Dye secondary antibodies (1:20.000, LI-COR Biosciences, Lincoln, NE, USA) and subsequently washed 4 *×* 5min in TTBS, 1 *×* 5min in TBS. The membrane was immediately scanned for protein bands using the 680nm and 780 nm channels at a scanning intensity of 4. Immunocomplexes were visualized using the Odyssey Infrared Imaging System (LI-COR). The following primary antibodies were used: α-Actin (sc-1616), α-Bax (N-20)(sc-493), α-Bcl-2 (C-2)(sc-7382), α-BclX_S_/_L_ (S-18)(sc-634), α-GAPDH (V-18)(sc-20357) (Santa Cruz, CA, USA), α-phospho-p53^Ser15^(cat.9284), α-phospho-p53^Ser46^ (cat.2521) (Cell Signaling Technology, Beverly, MA, USA) and α-p21^Waf1^ (cat.556430) (BD Pharmingen^™^, Becton-Dickinson, San Jose, California, USA). Antigen/primary antibody complexes were detected with near infrared-fluorescence-labeled, IR-Dye 800CW or IR-Dye 680RD, secondary antibodies (LI-COR Biosciences, Lincoln, NE, USA).

Alternatively, after electrophoresis the separated proteins were transferred to a PVDF membrane (Bio-Rad, Hercules, CA, USA). Blots were blocked 1 h at room temperature in TTBS containing low-fat powdered milk (5%). Incubations with primary antibodies were performed at 4°C for 12 h in blocking buffer (3% skim milk in TTBS). The membranes were then incubated with the corresponding counter-antibody and the proteins evidenced by enhanced chemiluminescence detection (SuperSignal West Femto System, Thermo Scientific, Rockford, IL, USA). The following primary antibodies were used: α-PARP (sc-8007) and α-Actin (sc-1616) (Santa Cruz Biotechnology, Santa Cruz, CA, USA), α-active Caspase-3 (ab13847) (Abcam Inc., Cambridge, MA, USA) and α-caspase-9 (cat. 9502) (Cell Signaling Technology, Beverly, MA, USA). The following secondary antibodies were used: a horseradish peroxidase-conjugated α-rabbit IgG; α-mouse IgG or α-goat IgG.

### Cell viability assay

Cells were plated onto Matrigel^™^ coated 96-well tissue culture plates at densities between 1x10^4^ and 3x10^4^ cells per well and grown until confluence. At the indicated time points post CPT (1μM for 3 h) and/or ABT-263 (0.1μM), WEHI-539 (1μM) or ABT-199 (1μM) removal, 50 μg/well of activated 2,3-bis (2-methoxy-4-nitro-5-sulfophenyl)-5 [(phenylamino) carbonyl]-2 H-tetrazolium hydroxide (XTT) (Sigma, St. Louis, MO, USA) in PBS containing 0.3 μg/well of the intermediate electron carrier, N-methyl dibenzopyrazine methyl sulfate (PMS) (Sigma, St. Louis, MO, USA) were added (final volume 100 μl) and incubated for 2–3 h at 37°C. Cellular metabolic activity was determined by measuring the absorbance of the samples with a multiplate spectrophotometer (Benchmark, Bio-Rad, Hercules, CA, USA) at a wavelength of 450 nm and subtracting the background absorbance at 690 nm.

### Assessment of DNA fragmentation

Apoptosis was quantified by direct determination of nucleosomal DNA fragmentation with Cell Death Detection ELISA^Plus^ kit (Roche, Mannheim, Germany). This assay uses specific monoclonal antibodies directed against histones from fragmented DNA, allowing the determination of mono and oligonucleosomes in the cytoplasmic fraction of cell lysates. Briefly, 2 × 10^5^ cells were plated on 24-well plates in 500 μl of culture medium. Fifteen hours after ABT-263 (0.1 μM) or ABT-199 (1 μM) addition or 3 h after CPT withdrawal (1 μM for 3 h), cells were lysed according to the manufacturer's manual, followed by centrifugation (200 x g, 5 min). When specified ABT-263 (0.1μM) or ABT-199 (1 μM) was added to the medium 1 h before and during treatment with CPT (1 μM for 3h). The mono and oligonucleosomes in the supernatants were determined using an anti-histone-biotin antibody. The resulting color development, which was proportional to the amount of nucleosomes captured in the antibody sandwich, was measured at 405 nm wavelength using a Benchmark microtiter plate reader (Bio-Rad Hercules, CA, USA). Results were expressed as DNA oligomers percentage, calculated from the ratio of absorbance of treated samples to that of the untreated ones.

### Statistical analysis

All of the results are expressed as the mean±SEM. The student's paired t test was used to determine significant differences between means, and P values below 0.05 were considered to be statistically significant.

## Results

### DNA damage-response signaling in CPT-treated hiPSCs and hESCs-derived NP

**Fig 1:**
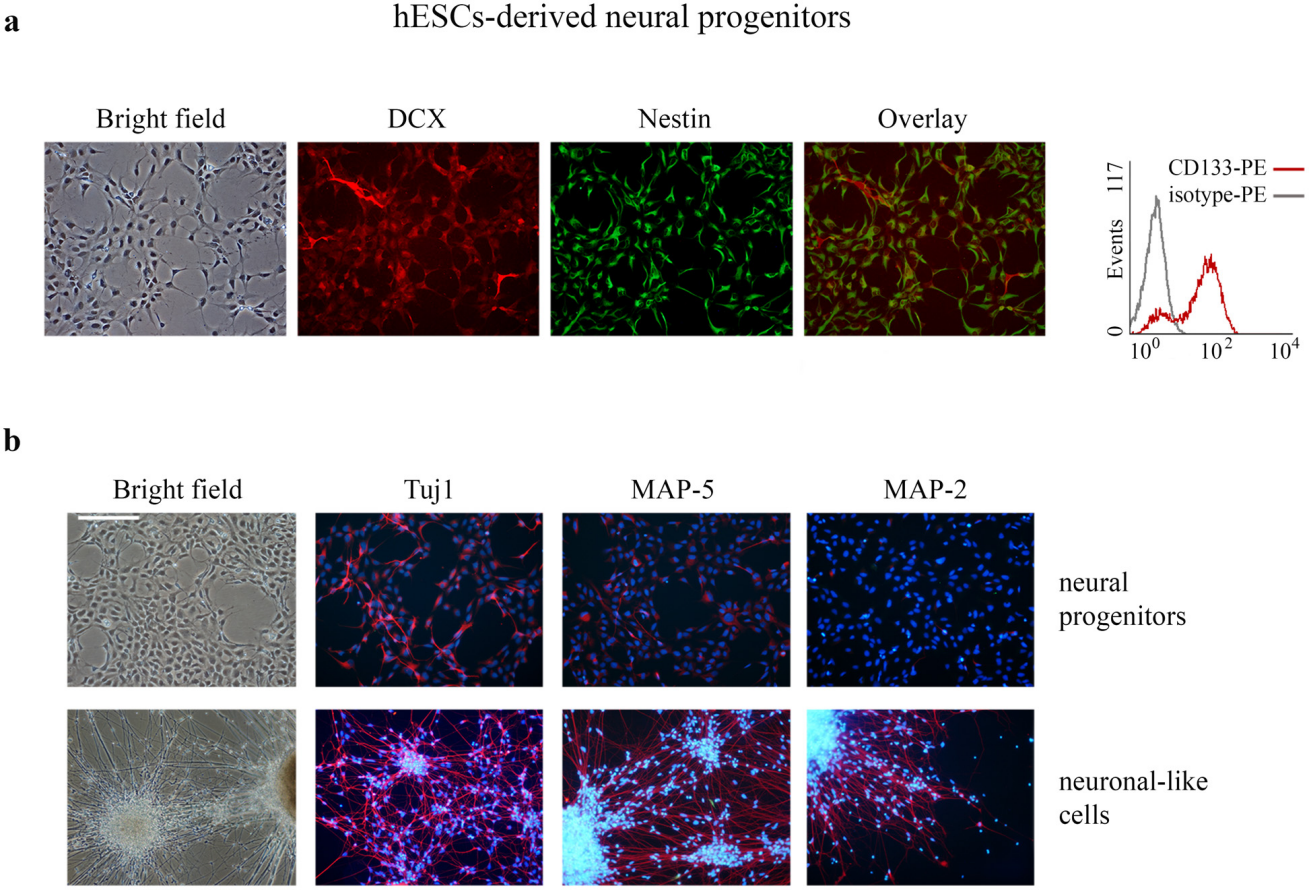
hESCs-derived NP and differentiated-neuronal-like cells phenotypic characterization. (a) Representative images of NP stained with primary antibodies against nestin and DCX (left panel). Representative flow cytometry histograms overlays of H9 hESCs-derived NP are shown to visualize CD133 expression relative to isotype control (right panel) (b) Representative images of NP and differentiated neuronal-like counterparts stained with primary antibodies against MAP-2, MAP-5 and Tuj1. The nuclei were counterstained with DAPI. The scale bars represent 100 μm.

Although recent progresses in stem cells biology fostered the characterization of hESCs responses to diverse genotoxic stresses, these are still far from being understood in full detail. Thus, to gain insight into the responses achieved by pluripotent cells upon DNA damage, we sought to compare how two different types of human pluripotent stem cells (hESCs and hiPSCs) respond to the DSBs inducing agent CPT, a well known inhibitor of DNA-topoisomerase I complexes. Taking into account that hESCs offer an accessible and manageable platform to model the early stages of human development, we generated hESCs-derived NP to further characterize the cellular responses triggered by genotoxic stress in hESCs undergoing neural differentiation. To do so, we subjected H9 hESCs to a neural differentiation protocol. We determined that the hESCs-derived NP express the stemness markers nestin and CD133 and the neuronal migration protein, doublecortin (DCX) (Fig 1a). These NP further differentiate into process-bearing neuronal-like cells that stain positively for microtubule-associated proteins MAP-2 and MAP-5 and neuron-specific class III β-tubulin (Tuj1) (Fig 1b, bottom panel).

In a previous study we have characterized the response of H9 hESCs to DNA damage induced by CPT [26], herein we compared their behavior with that of FN2.1 hiPSCs line [25] and differentiated cells from the same cellular background, placing especial emphasis on the mechanisms underlying survival/death decisions.

**Fig 2:**
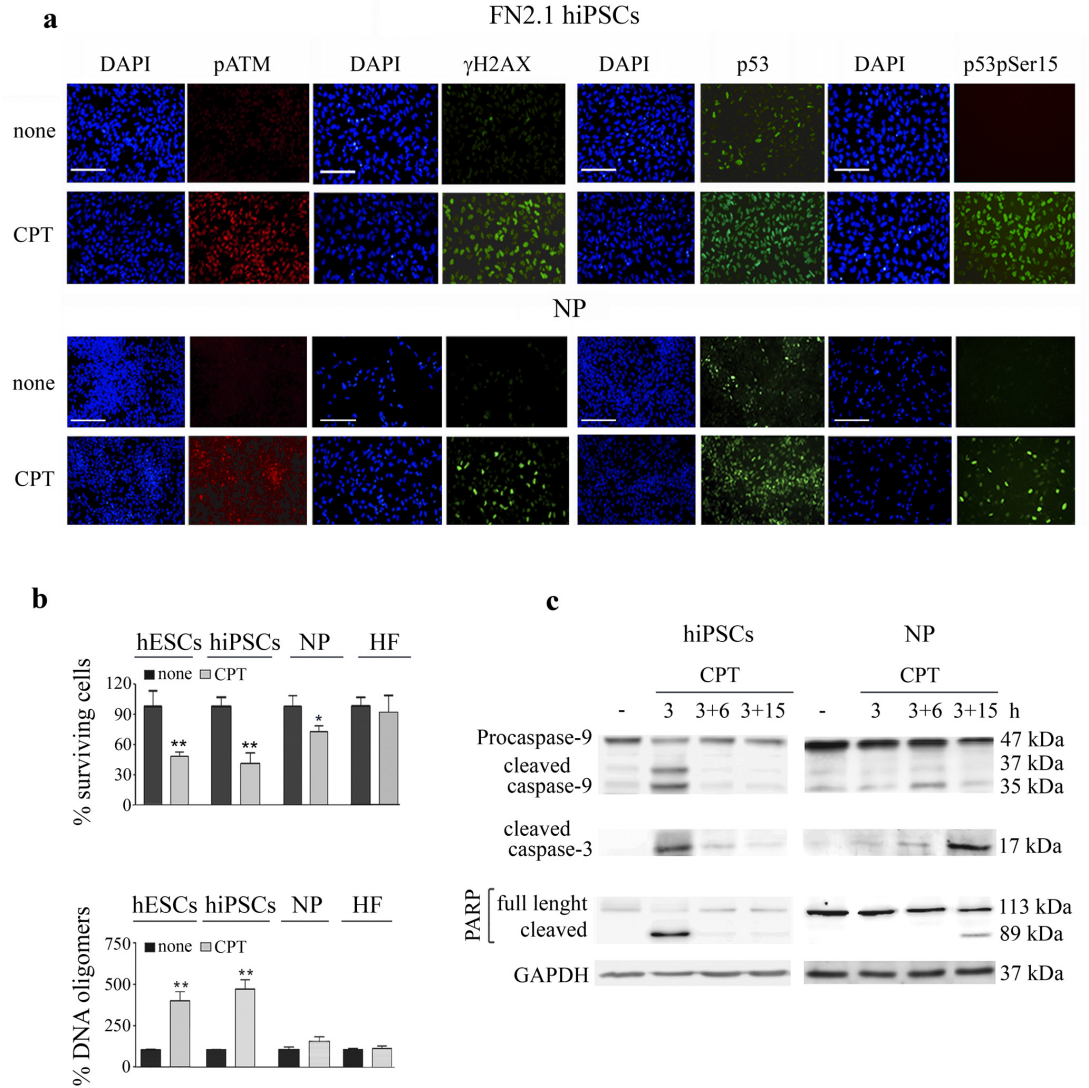
CPT activates DNA damage response and triggers apoptosis in hiPSCs and hESCs-derived NP. (a) Immunofluorescence photomicrographs of genotoxic-treated (1μM during 3 h) hiPSCs and NP performed immediately after CPT treatment (1μM during 3 h). The figure shows representative images of cells stained with primary antibodies against ATM phospho-serine1981 (pATM), histoneγH2AX, p53, p53 phospho serine 15 (p53pSer15). Nuclei were counterstained with DAPI. The scale bars represent 100 μm. (b) Cell viability was measured in hESCs, hiPSCs, NP and HF treated or not with 1 μM CPT during 3h by the XTT/PMS assay 6 h after drug removal (top panel). Results are presented as the percentage of the viability of untreated cells. Each bar represents the mean±SEM of three independent experiments performed in quintuplicate. A paired Student’s *t* test was used to compare CPT treated samples to untreated controls. After a withdrawal period of 3 h CPT-treated cells were harvested, and DNA oligomers were quantified by immunoassay (bottom panel). Results are presented as the percentage of DNA oligomers of untreated cells. Each bar represents the mean±SEM of three independent experiments performed in triplicate. *P< 0.05, **P< 0.001. (c) Time course of caspase-9, caspase-3 and PARP cleavages in hiPSCs and NP upon CPT treatment were analyzed by Western blotting with anti-caspase-9, anti-cleaved-caspase-3 and anti-PARP specific antibodies, immediately after (3 h), 6 h or 15 h post drug removal (3+6, 3+15, respectively). GAPDH was used as loading control.

Similarly to what occurred in hESCs, we found that CPT treatment (1μM for 3 h) led to concurrent phosphorylation of ATM (serine1981) and H2AX (serine139, γH2AX) in FN2.1 hiPSCs and NP. Nuclear accumulation and phosphorylation of p53 on serine 15 was also observed within 3 h in damaged cells (Fig 2a).

We then evaluated the effect of CPT (1μM for 3 h) on H9 hESCs, FN2.1 hiPSCs, NP and HF viability. To do so, we determined the percentage of surviving cells after a withdrawal period of 6 h using a XTT/PMS vital dye assay. We found that CPT has a greater effect on hiPSCs viability than in NP, with the survival of cells declining to approximately 43% and 70%, respectively (Fig 2b). Importantly, both pluripotent stem cell types resulted equally sensitive to topoisomerase I inhibition. Notably, the HF used for the hiPSCs reprogramming resulted resistant to CPT action at both the concentration and time frame tested.

To elucidate if the different susceptibility to CPT may be associated to the percentage of cells residing in S phase in each cell type we characterized their cell cycle distribution by flow cytometric analysis of DNA content. Flow cytometry results revealed that, similar to that of H9 hESCs, a substantial fraction of FN2.1 hiPSCs cells (65%) progresses through S phase at any given time. In contrast, NP and HF exhibited a small percentage of cells in S phase (14% and 10%, respectively) (S1 Fig). Thus, if we take into account that the primary mechanism by which CPT kills cells is through S phase-dependent cytotoxicity, the higher decrease in hiPSCs viability observed upon CPT treatment may be attributed, at least in part, to the elevated percentage of pluripotent cells going through the S phase of the cell cycle.

To further investigate if the loss of cell viability was due to CPT-induced apoptosis, we measured DNA fragmentation (cytoplasmic oligonucleosomal fragments), a late event in the apoptotic cascade and one upon which a variety of caspase-dependent and independent pathways converge [27]. To do so, we quantified the abundance of DNA fragments with an enzyme-linked immunosorbent assay (ELISA) in treated and untreated cells. As shown in Fig 2b (bottom panel) a dramatic increase in the percentage of DNA oligomers was observed 3 h after CPT removal (1 μM for 3 h) in damaged pluripotent cells (hESCs and hiPSCs). In contrast only a small not significant increase in DNA fragmentation was observed in damaged hESCs-derived NP (Fig 2b). Remarkably, no appreciable differences appeared between the amounts of DNA oligomers present in stressed and unstressed HF as judged by ELISA quantification (Fig 2b, bottom panel).

Caspases, a family of cysteine proteases, act as common death effector molecules, which upon activation cleave various substrates in the cytoplasm or nucleus. Therefore we decided to evaluate whether topoisomerase I inhibition leads to caspases activation in hiPSCs. To this end, we performed Western blot analysis and found that 3 h after CPT treatment initiator pro-caspase-9 (47 kDa) was processed into active fragments (37/35 kDa) (Fig 2c, left upper panel). Moreover, using a specific antibody that recognizes cleaved caspase-3 we determined that this effector caspase was also activated 3 h after CPT addition (Fig 2c, left middle panel). Active executioner caspase-3 can further cleave downstream substrates involved in apoptotic changes such as PARP. We found that hiPSCs accumulate cleaved PARP within 3 h after DNA damage (Fig 2c, left bottom panel). Interestingly, we determined that upon CPT exposure FN2.1 hiPSCs and H9 hESCs show comparable kinetics of caspase-9 and caspase-3 activation [26].

Next, we wondered whether there are any differences between the apoptotic responses induced by topoisomerase I inhibition in undifferentiated hESCs and hESCs undergoing neural differentiation. In this regard, we observed that in hESC-derived NP CPT triggers proteolytic processing of pro-caspase-9, demonstrated by the appearance of 35kDa cleaved fragment 6 h after drug withdrawal (Fig 2c, right upper panel). Caspase-9 activation was followed by caspase-3 and PARP cleavage which became appreciable 15 h after genotoxic removal (Fig 2c, right middle and bottom panels). Altogether these findings revealed that CPT appears to induce similar responses but to different extents and with divergent kinetics in hESCs, hiPSCs and hESCs-derived progeny.

### p53 undergoes phosphorylation at serine 46 upon CPT exposure in pluripotent stem cells and NP

**Fig 3:**
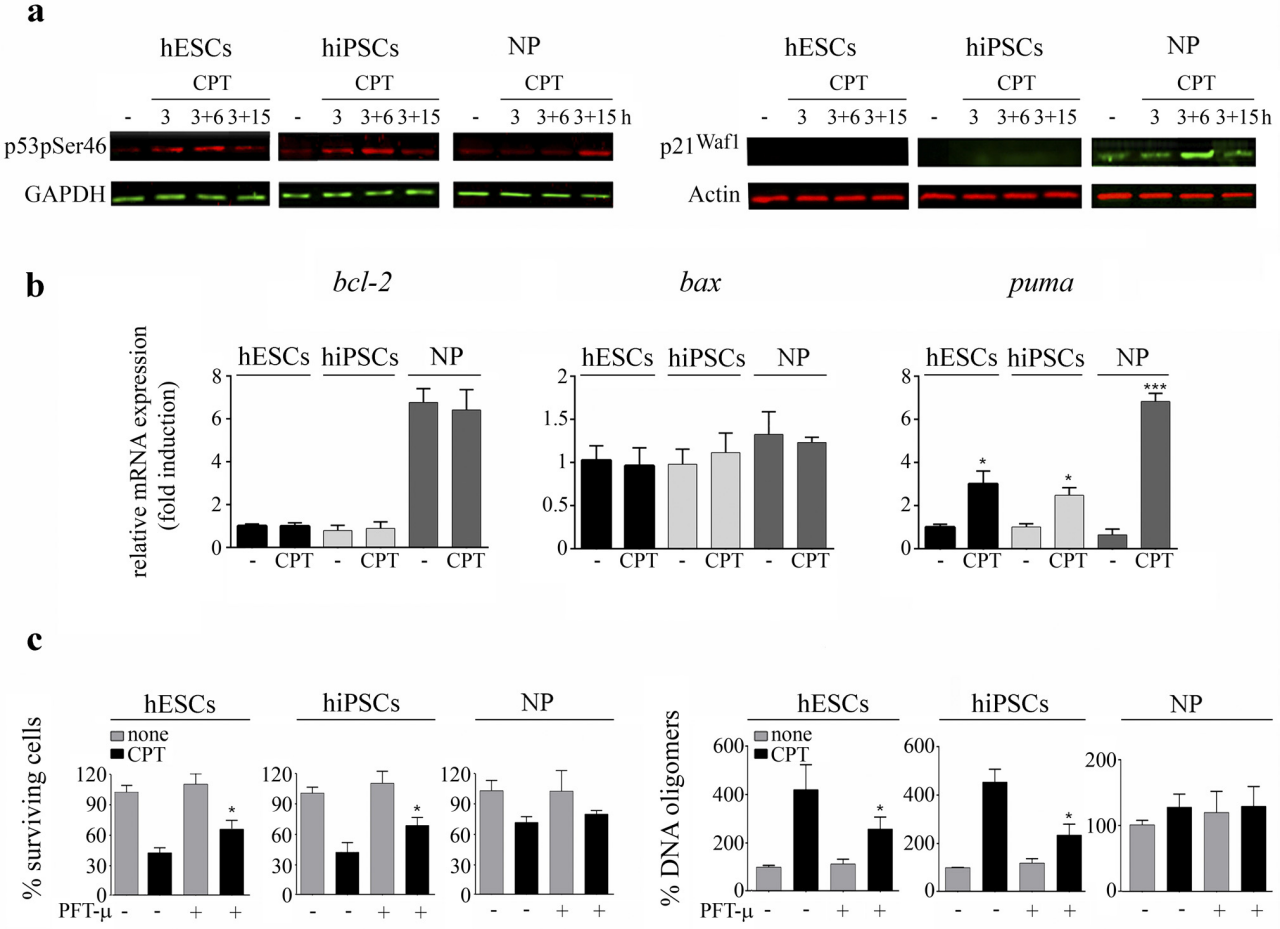
CPT triggers p53 phosphorylation at Serine 46. (a) Time course of p53 phosphorylation at serine 46 upon CPT treatment was analyzed by Western blotting using anti-phospho p53 Ser46 (p53pSer46) specific antibody, immediately after (3h), 6h or 15 h post drug removal (3+6 h, 3+15 h, respectively). GAPDH was used as loading control (left panel). p21^Waf1^ expression levels upon CPT exposure in hiPSCs and hESCs-derived NP were analyzed by Western blotting immediately after 1μM CPT treatment (3h), 6h or 15h post genotoxic removal (3+6 h, 3+15 h, respectively) using specific antibodies against p21^Waf1^ and Actin (right panel) (b) mRNA levels of *bcl-2*, *bax* and *puma* were analyzed by Real Time RT-PCR in CPT treated or untreated hESCs, hiPSCs and NP. GAPDH expression was used as normalizer. Graph shows mRNA fold induction relative to undamaged hESCs arbitrarily set as 1. Each bar represents the mean ± SEM of three independent experiments. In all cases, a paired Student's t test was used to compare CPT-treated samples to untreated ones (c) Cell viability was measured by the XTT/PMS assay at 6 h after drug removal in PFT-μ treated or untreated cells (10 μM, 1 h before and during CPT exposure). Results are presented as the percentage of the viability of untreated cells (left panel). PFT-μ treated or untreated cells were harvested 3h after CPT treatment (1 μM over a 3 h period), and DNA oligomers were measured by immunoassay. Results are presented as the percentage of DNA oligomers of untreated cells. Each bar represents the mean ± SEM of three independent experiments performed in triplicate (right panel) In all cases, a paired Student's t test was used to compare CPT plus PFT-μ treated samples to CPT treated ones.*P< 0.05, ***P< 0.0001.

In response to DNA damage p53 undergoes a series of post-translational modifications and some of them are thought to play a role in target gene selectivity [28]. In this respect, recent studies suggest that phosphorylation of p53 on serine 46 is an important cell fate determinant favoring p53-dependent transcriptional activation of pro-apoptotic target genes [29, 30]. These considerations prompted us to explore whether p53 undergoes phosphorylation on serine 46 in pluripotent stem cells and hESCs-derived NP cells exposed to CPT. To address thisquestion, CPT-treated (1 μM for 3 h) H9 hESCs, FN2.1 hiPSCs and NP were harvested at different time points post-treatment and subjected to Western blot assays. Immunoblot analysis revealed that p53 is phosphorylated on serine 46 upon CPT exposure in all tested cell types. Enhanced levels of phospho-p53 (Ser46) were seen 6 h after CPT withdrawal in hESCs and hiPSCs. However, in NP this post-translational modification became evident only 15 h after drug removal (Fig 3a, left panel). The fact that phosphorylation of p53 on serine15 preceded phosphorylation on serine 46 agrees with previous findings describing that specific phosphorylation events may be dependent on previous post-translational modifications of p53. In this sense, it has been suggested that phosphorylation of p53 on serine 15 by ATM promotes further phosphorylation on serine 6, serine 9, threonine 18, serine 20 and serine 46 [31].

In a previous study the absence of detectable levels of p21^Waf1^ protein in H9 hESCs despite the stabilization of p53 triggered by CPT was confirmed by Western blot analysis [26]. Therefore, we asked whether this cyclin dependent kinase inhibitor would also be absent in undamaged and damaged hiPSCs. To answer this question we subjected untreated and CPT-treated hiPSCs to Western blot analysis. We found that similarly to what occurred in hESCs, this pluripotent cell type does not exhibit detectable levels of p21^Waf1^ even after severe DNA damage. In contrast, we determined that hESCs undergoing neuronal differentiation express perceptible levels of p21^Waf1^ which were robustly induced 6 h after genotoxic withdrawal (Fig 3a, right panels). This induction was previously observed in CPT-treated embryoid bodies (H9 hESCs undergoing serum-induced differentiation) [26].

p53 as a transcription factor responds to certain cellular stressors by inducing transcriptional programs that can lead to growth arrest or apoptosis. p53 mediates arrest by transcriptionally activating genes such as p21^Waf1^, whereas p53-dependent apoptosis is triggered by transactivation of pro-apoptotic genes, such as Bax and Puma [32]. In addition to its ability to promote the transcription of pro-apoptotic Bcl-2 family members, in some settings p53 could regulate apoptosis by direct repression of pro-survival Bcl-2 transcription [33]. To address whether p53 stabilization was accompanied by changes in *bcl-2*, *bax* and *puma* mRNA levels we performed real time quantitative RT-PCR analysis in damaged and undamaged cells. s We observed a strong induction of *puma* mRNA 6h after CPT withdrawal (1μM for 3 h) in all tested cell lines, while no appreciable changes in *bcl-2* and *bax* mRNAexpression levels were detected (Fig 3b). Interestingly, we found that hESCs and hiPSCs exhibit significantly lower *bcl-2* mRNA levels than NP (Fig 3b left panel).

### p53's mitochondrial death program regulates the extent of genotoxic-induced cell death in pluripotent cells

In several cell types p53 mounts a direct mitochondrial death program in response to intense stress [34]. To elucidate whether this program is also functional in hiPSCs and hESCs-derived progeny we exposed cells to PFT-μ, a small molecule that inhibits p53 binding to mitochondria by reducing its affinity to Bcl-xL and Bcl-2 but has no effect on p53-dependent transactivation [35, 36]. To this end, we performed cell viability assays 6 h after drug removal in CPT-treated or untreated cells in the presence of PFT-μ (10μM, 1 h before and during CPT exposure). By XTT/PMS assays we determined that cell viability was increased in damaged pluripotent cells when PFT-μ was added (22% in hESCs and 27% in hiPSCs) (Fig 3c, left panel). Interestingly, we also found that as previously described in hESCs [26], PFT-μ protects hiPSCs from spontaneous apoptosis (12 to 16%). Conversely, no appreciable differences were observed in hESC-derived NP viability in the presence of this inhibitor (Fig 3c, left panel). To further gain insight into the involvement of p53 in CPT-induced cell death in these cell types, we determined apoptosis levels by quantifying the percentages of cytosolic DNA oligomers present in CPT-treated hESCs, hiPSCs and NP in the presence or absence of PFT-μ. As seen in Fig 3c (right panel), PFT-μ treatment significantly reduced the amount of DNA oligomers that were present in the cytoplasm of pluripotent stem cells 3h after genotoxic removal (41% in hESCs and 48% in hiPSCs), while no significant changes were observed in NP. These results suggest that as occurs in several cell types, p53's mitochondrial death program regulates the extent of genotoxic-induced cell death and particularly in hESCs and hiPSCs also influences spontaneous cell death.

### Pluripotent cells show lower levels of anti-apoptotic Bcl-2 than hESCs-derived neural progenitors

**Fig 4:**
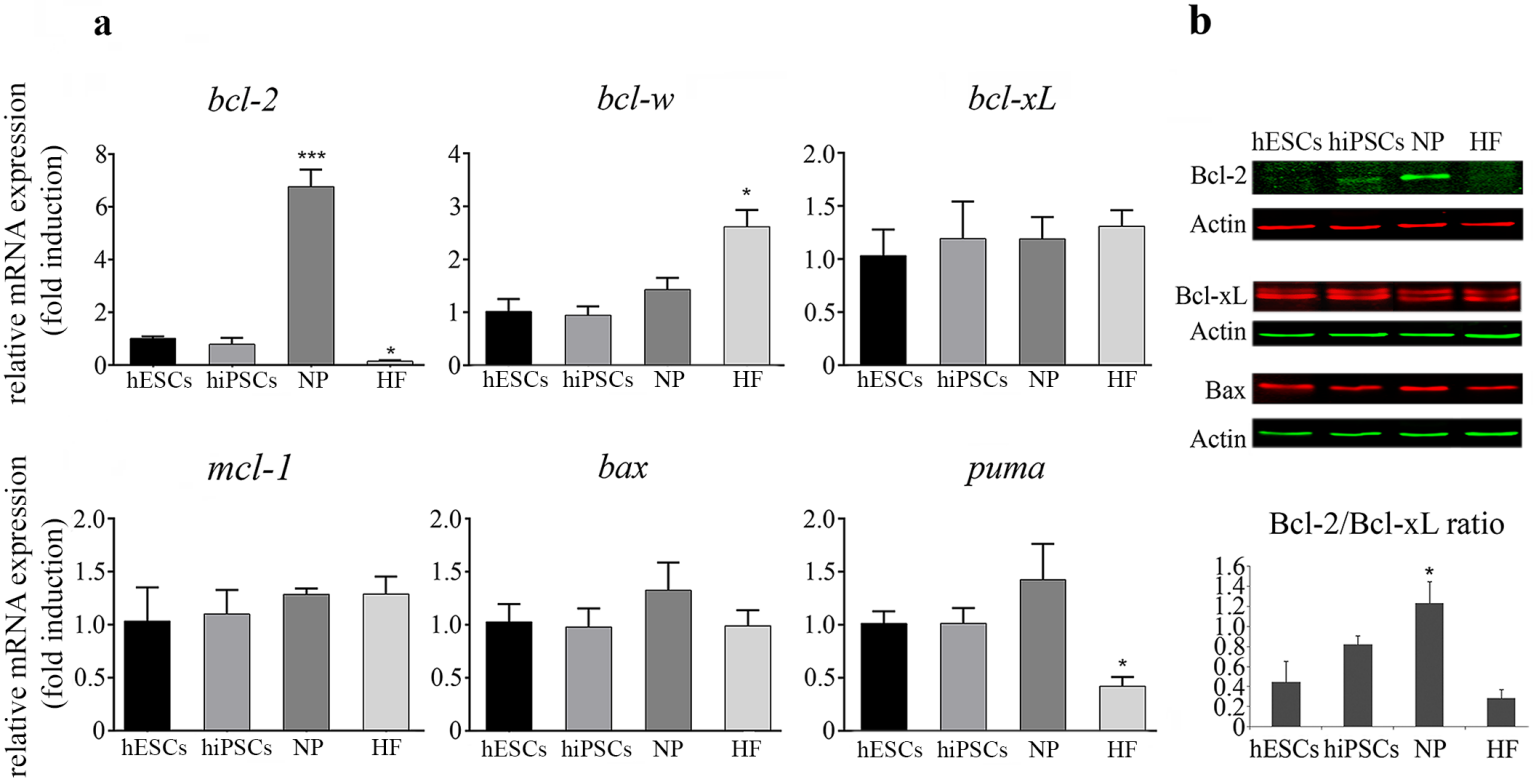
Bcl-2 family members expression profile in hESCs, hiPSCs, hESCs-derived NP and HF. (a) mRNA expression levels of *bcl-2*,*bcl-w*,*bcl-xL*,*mcl-1*,*bax* and *puma* were analyzed by Real Time quantitative RT-PCR in hESCs, hiPSCs, NP and HF. GAPDH expression was used as normalizer. Graph shows mRNA fold induction relative to hESCs arbitrarily set as 1. The mean ± S.E. from three independent experiments are shown. In all cases, a paired Student's t test was used to test for significant differences between hESCs and each cell line tested *P < 0.05, ***P< 0.0001. (b) Representative Western blot images are shown. Cellular extracts were prepared and Western blot analyses were carried out using anti-Bcl-2, anti-Bcl-xL and anti-Bax specific antibodies. Actin served as loading control (top panel). Intensities of protein bands corresponding to Bcl-2 and Bcl-xL were measured using computer-assisted densitometric analysis and normalized to the intensity of Actin. Intensities of Bcl-2 and Bcl-xL were also compared to each other and presented as the average ratio for each cell line (bottom panel). A paired Student's t test was performed to test for significant differences between hESCs and each cell line tested *P < 0.05.

Frequently, the ability to achieve the apoptotic threshold is predetermined by the endogenous expression levels of critical pro- and anti-apoptotic factors. Thus, to assess the expression levels of key regulators of apoptosis in pluripotent and progenitor cells, we performed real time quantitative RT-PCR analysis. We determined that undifferentiated and differentiated cells display similar expression profiles of the anti-apoptotic factors *bcl-w*, *bcl-xL*, and *mcl-1* and the pro-apoptotic proteins *bax* and *puma* (Fig 4a). However, we found that hESCs and hiPSCs exhibit lower levels of pro-survival factor *bcl-2* mRNA than NP (Fig 4a). Furthermore, using Western blotting we observed a strong correlation between *bcl-2*, *bcl-xL* and *bax* mRNAs and their protein products (Fig 4b). Importantly, this expression profile of Bcl-2 was previously observed in WA01 hESC line when comparing undifferentiated cells with their all-*trans* retinoic acid-differentiated counterparts [16], supporting the concept that the intrinsic balance of pro-apoptotic and anti-apoptotic proteins is closer to the apoptotic threshold in undifferentiated cells than in differentiated ones. In parallel, we examined the expression profile of the aforementioned pro- and anti-apoptotic factors in HF. Real-time PCR data showed that these cells display lower levels of *puma* mRNA and higher levels of *bcl-w* mRNA than pluripotent and NP cells. Interestingly, we found that HF exhibit very low levels of Bcl-2. This reduced expression pattern of Bcl-2 has been previously observed during *in vitro* aging of normal fibroblasts [37].

### The BH3-mimetic drug, ABT-263, triggers apoptosis and exacerbates CPT-induced cell death in hESCs, hiPSCs and NP

At the onset of apoptosis cells typically activate different species of BH3-only proteins, which preferentially bind to and inactivate the excess numbers of anti-apoptotic Bcl-2 family proteins. Recent studies indicate that all anti-apoptotic molecules must be sequestered before cell death can occur [38]. Thus, the threshold for apoptosis may be determined in large part by the proportion of pro-survival relatives that remain unoccupied by BH3-only proteins.

These considerations prompted us to ask if disturbing the balance between pro- and anti-apoptotic proteins would enable cells to activate apoptosis. To answer this question, we exposed cells to the small molecule BH3-protein mimetic, ABT-263 [21], to investigate whether increased occupancy of these BH3-binding pockets is in itself sufficient to initiate a cell death response in pluripotent and NP cells.

**Fig 5:**
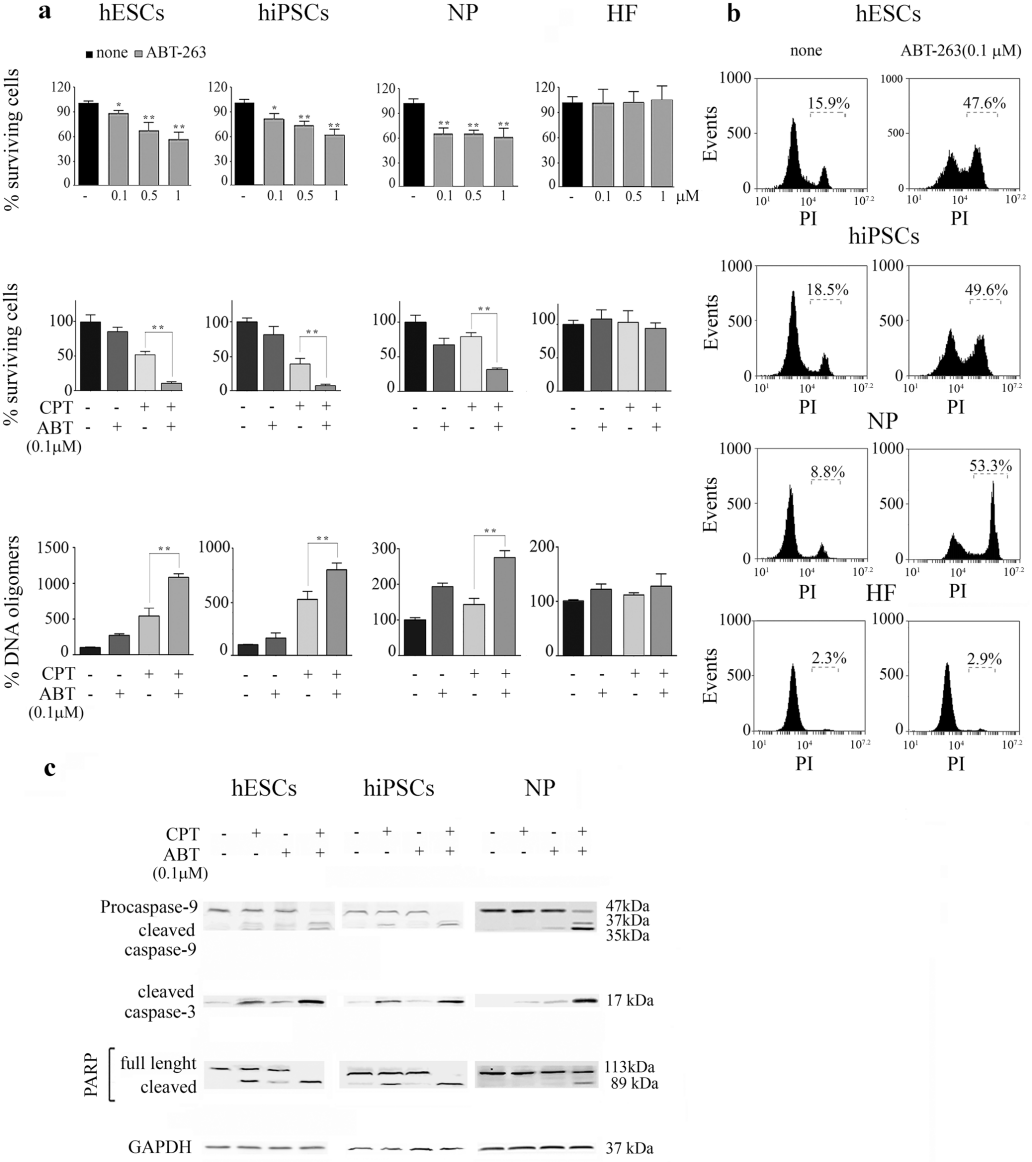
ABT-263 reduces cell viability and potentiates CPT-induced apoptosis in hESCs, hiPSCs and NP. (a) H9 hESCs, FN2.1 hiPSCs, hESCs-derived NP and HF were treated with increasing concentrations of ABT-263 (0.1–1 μM) during 15h (top panel) or pre-treated with ABT-263 (0.1μM) for 1hour and then treated with CPT (1μM during 3h) (middle panel). Cell viability was measured by the XTT/PMS assay 15 h post ABT-263 (0.1μM) addition or 6 h after ABT-263 (0.1μM) and/or genotoxic removal. Results are presented as the percentage of the viability of untreated cells. Each bar represents the mean±SEM of three independent experiments performed in quintuplicate. After 15 h of ABT-263 (0.1μM) addition or a withdrawal period of 3 h ABT-263 (0.1μM) and/or CPT-treated cells were harvested, and DNA oligomers were quantified by immunoassay (bottom panel). Results are presented as the percentage of DNA oligomers of untreated cells. Each bar represents the mean±SEM of three independent experiments performed in triplicate. A paired Student's t test was used to compare ABT-263-treated samples with untreated ones (top panel) or CPT plus ABT-263 treated samples to CPT treated ones (middle and bottom panel)*P< 0.05, **P< 0.001. (b) Representative histograms of PI-stained ABT-263 (0.1μM) treated cells over a 20 h period or untreated unfixed cells. Percentage of PI positive cells (late apoptotic or necrotic) was determined by flow cytometric analysis (c) Caspase-9, caspase-3 activation and PARP cleavage in hESCs, hiPSCs and NP upon CPT (1μM for 3h), ABT-263 (0.1μM for 15 h) or ABT-263 (0.1μM) for 1 h prior and during CPT treatment were analyzed by Western blotting 3 h post genotoxic removal. GAPDH was used as loading control.

To evaluate whether ABT-263 triggers similar effects in hESCs, hiPSCs, NP and in the starting cell population (HF) used to generate hiPSCs, we exposed these cell types to increasing concentrations of this drug (0.1μM to 1 μM) for 15h. As shown in Fig 5a, ABT-263 treatment caused a concentration-dependent death in pluripotent cells. ABT-263 (0.1 μM) exposure resulted in a mild, but statistically significant, drop in viability for both hESCs (13%) and hiPSCs (16.5%) (Fig 5a, top panel). In contrast, ABT-263 (0.1μM) elicited a marked decrease in NP viability (36%). Our results indicate that NP display a higher sensitivity to ABT-263 than pluripotent cells while HF are almost totally insensitive to this molecule.

The differential cytotoxicity of ABT-263 (0.1μM) in the tested cell types was further demonstrated 20 h after drug addition by flow cytometry analysis with PI staining. PI is membrane impermeant and does not enter viable cells with intact membranes, therefore the histograms in Fig 5b show the percentages of cells whose plasma membrane integrity is comprised (late apoptosis or necrosis). This differential degree of susceptibility to ABT-263 might be explained, at least in part, by the different expression levels of Bcl-2 displayed by pluripotent and NP cells.

In undifferentiated hESCs and hiPSCs there is a biased expression of many pro-apoptotic genes, against relatively fewer anti-apoptotic ones [16, 39]. This expression profile suggests that the survival of these pluripotent cells may be highly regulated by the availability of functional anti-apoptotic factors. Therefore, we wondered whether the activities of anti-apoptotic factors may play important roles in pluripotent cells fate upon genotoxic stress. In order to address this issue, we pre-treated cells with ABT-263 and exposed them to CPT. Cell viability assays measured 6 h after genotoxic removal revealed that ABT-263 addition dramatically increases hypersensitivity to CPT of hESCs and hiPSCs lines (Fig 5a middle panel). Moreover, hESCs undergoing neural differentiation dramatically increased their sensitivity to DSB-induced cell death when the activities of Bcl-2 factors were impaired by ABT-263 treatment.

Additionally, we determined the effect of ABT-263 (0.1μM) on CPT-induced apoptosis, by measuring the extent of oligonucleosomal formation by ELISA after a withdrawal period of 3h. We found that ABT-263 (0.1 μM) treatment markedly augments the amount of CPT-induced DNA oligomers in H9 hESCs (~2.0 fold), FN2.1 hiPSCs (~1.6 fold) and NP (~2.0 fold) (Fig 5a, bottom panel). Moreover, DNA fragmentation and loss of cell viability was accompanied by typical features of apoptosis such as cell detachment and ballooning (S2 Fig). ABT-263 accelerated the appearance of these morphological changes in hESCs, hiPSCs and NP, which became evident as early as 3 h after CPT addition (compare ABT+CPT,1+3h with CPT, 3+12h) (S2 Fig). Contrarily, HF appear to have a higher apoptotic threshold and, as a result, do not undergo apoptosis in the presence of ABT-263 and CPT, at least at the concentrations and time frames tested (Fig 5 and S2 Fig).

Then, to further characterize the events which occurred as a result of the occupancy of the BH3-pockets by ABT-263 that precipitated the onset of programmed cell death, we assessed the expression of active caspase-9 and -3 as well as the presence of cleaved PARP by Western blot analysis. As shown in Fig 5c the presence of ABT-263 increased the abundance of active fragments of initiator and effector caspases and the extent of cleaved PARP in damaged pluripotent and progenitor cells. Moreover, in NP the appearance of activated caspases and the 89 kDa PARP cleavage fragment was evident as early as 3 h after genotoxic addition while in the absence of this BH3-mimetic molecule these phenomena became evident after a withdrawal period of 15 h (Fig 2c). These results agree with previous findings from other labs describing that ABT-263 treatment increased the susceptibility of differentiated hESCs to the D**NA** double-strand cleaving agent neocarzinostatin [16].

### Anti-Bcl-xL strategies (siRNA or BH3mimetic: WEHI-539) sensitize pluripotent and NP cells to CPT-induced DNA damage

**Fig 6:**
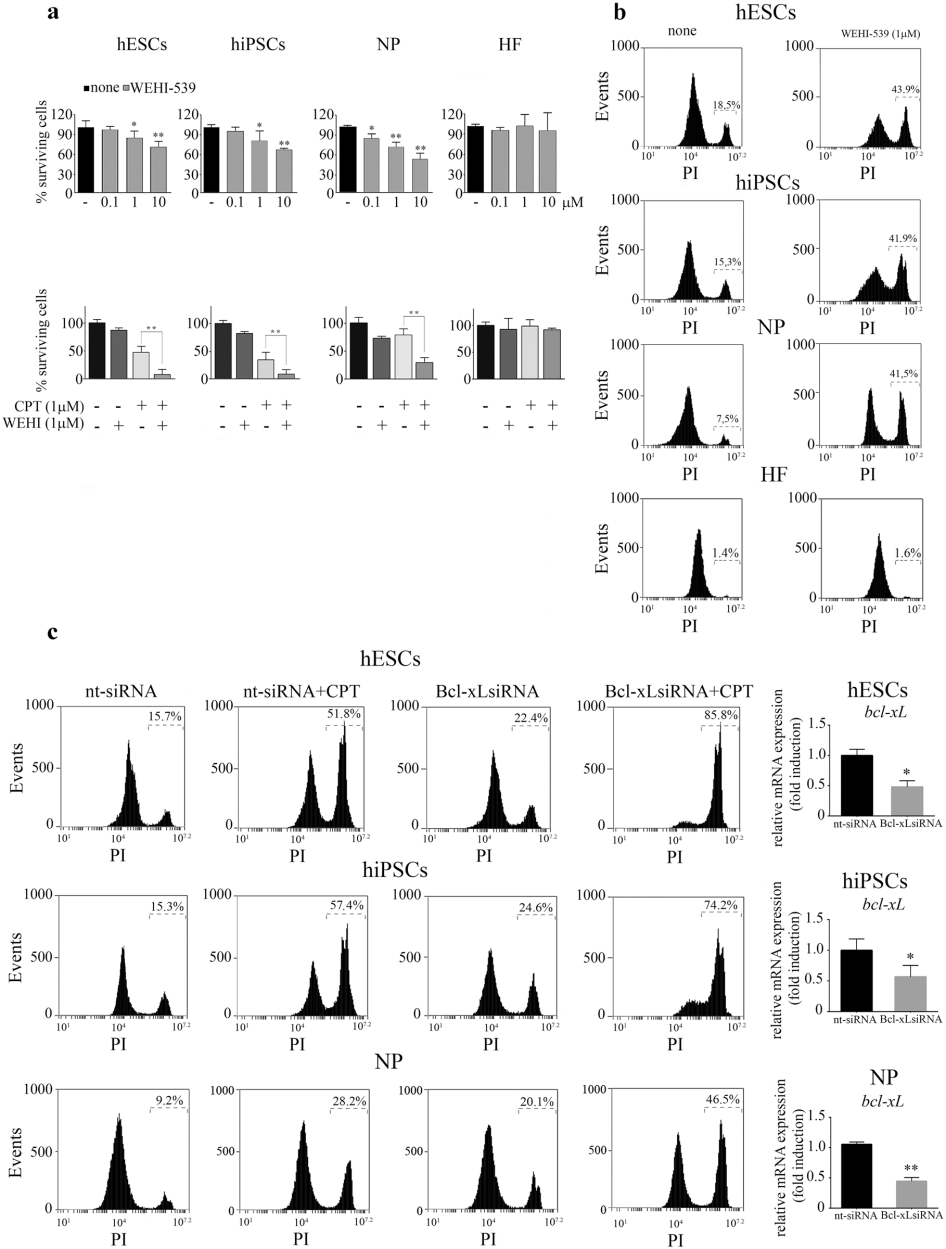
Pharmacological inhibition or siRNA-mediated down regulation of Bcl-xL sensitize hESCs, hiPSCs and NP to CPT induced DNA damage. (a) H9 hESCs, FN2.1 hiPSCs, hESCs-derived NP and HF were left untreated or treated with increasing concentrations of WEHI-539 (0.1–10 μM) during 15h (top panel) or treated with CPT (1μM) for 3h or pre-treated with WEHI-539 (1μM) for 1h r and then exposed to CPT (bottom panel). Cell viability was measured by the XTT/PMS assay 15 h post WEHI-539 (1μM) addition or 6 h after CPT removal. Results are presented as the percentage of the viability of untreated cells. Each bar represents the mean±SEM of three independent experiments performed in quintuplicate. A paired Student's t test was used to compare WEHI-539-treated samples with untreated ones (top panel) or CPT plus WEHI-539 treated samples to CPT treated ones (bottom panel) *P< 0.05, **P< 0.001. (b) Representative histograms of PI-stained WEHI-539 (1μM) treated cells over a 20 h period or untreated unfixed cells. Percentage of PI positive cells was determined by flow cytometric analysis (c) H9 hESCs, FN2.1 hiPSCs, hESCs-derived NP cells were transfected with nt-siRNA or Bcl-xL siRNA (50 nM) and exposed to CPT (1μM for 3h)48 h post-transfection. Representative histograms of PI-stained transfected cells 12 h after genotoxic removal (left panel). mRNA expression levels of *bcl-xL* in nt-siRNA and Bcl-xL siRNA transfected cell lines were analyzed by Real Time RT-PCR (right panel). GAPDH expression was used as normalizer. Graph shows mRNA fold induction relative to nt-siRNA transfectants arbitrarily set as 1. *P < 0.05 **P < 0.001.

To further investigate the relevance of Bcl-xL as a survival factor in pluripotent and NP cells we made use of a recently developed Bcl-xL selective BH3 mimetic, WEHI-539. We found that exposure of H9 hESCs and FN2.1 hiPSCs cells to this compound caused loss of viability at concentrations between 1–10 μM (Fig 6a top panel). Importantly, NP display a higher susceptibility to WEHI-539 treatment than pluripotent cells (Fig 6a and 6b). Moreover, as occurred in the presence of 0.1μM ABT-263, we determined that a suboptimal dose of 1 μM WEHI-539 effectively enhances the response to CPT in pluripotent and NP cells but shows no effect in HF (Fig 6a, bottom panel).

In order to gain insight into the involvement of Bcl-xL in the regulation of pluripotent and NP cells survival we used siRNA-induced gene silencing. To determine the effectiveness of siRNA mediated knockdown of Bcl-xL in transfected cells we performed real time RT-PCR of RNA from cells transfected with either non targeting control siRNA (nt-siRNA) or Bcl-xL specific siRNA. As shown in Fig 6c a significant decrease in Bcl-xL mRNA in cells transfected with Bcl-xL siRNA was observed. As expected, we found that siRNA-mediated downregulation of Bcl-xL markedly sensitized pluripotent and progenitor cells to genotoxicity induced by topoisomerase I inhibition (Fig 6c and S2 Fig). Downregulation of Bcl-xL expression by siRNA did not affect the morphology or adhesion of HF even after CPT treatment (S2b Fig). Taken together these results confirm that Bcl-xL represents an anti-apoptotic molecule of dominant physiological relevance for hESCs, hiPSCs and NP.

### Bcl-2 selective inhibition does not affect apoptotic levels triggered by CPT in pluripotent and NP cells

**Fig 7:**
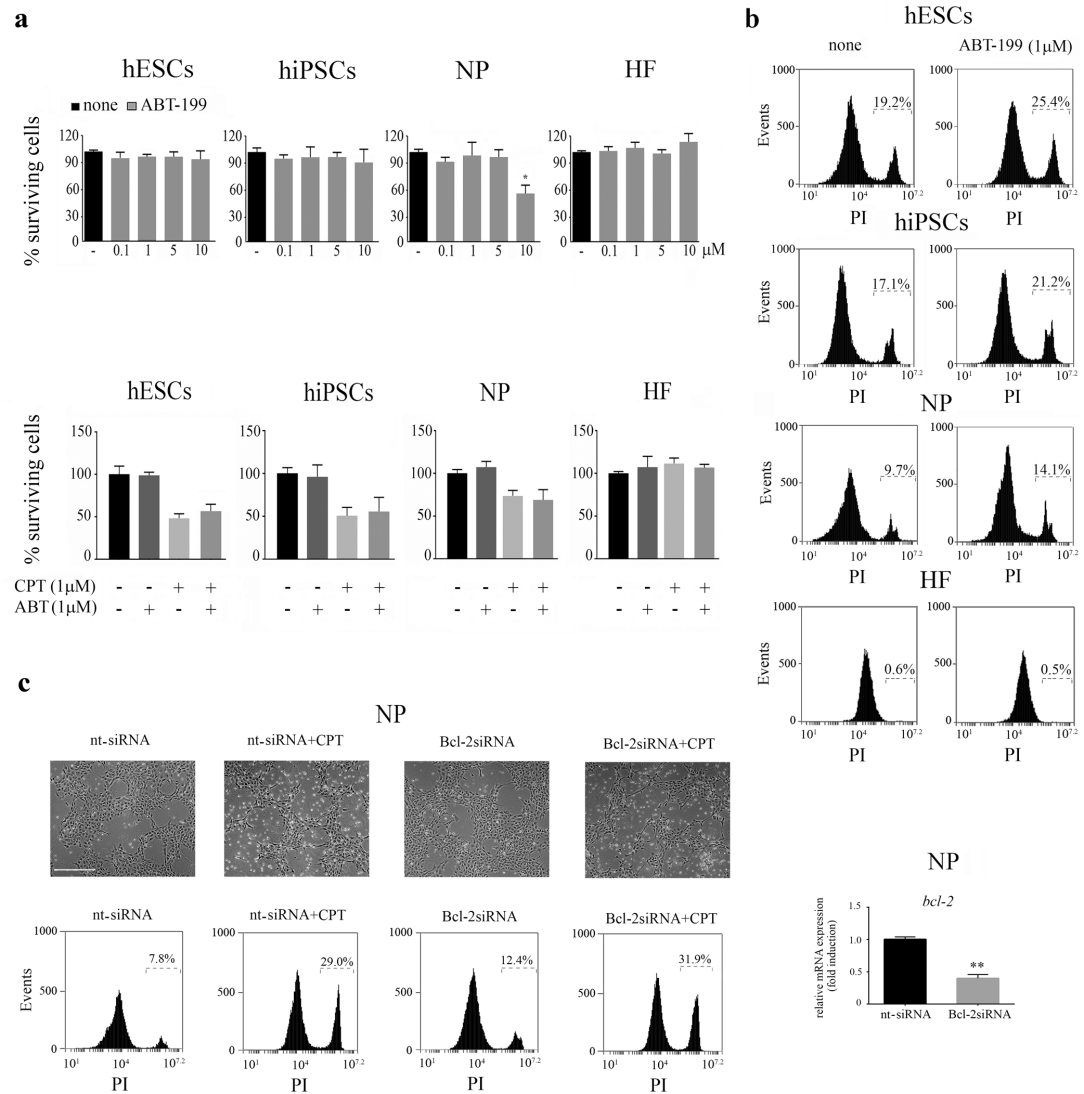
Effects of Bcl-2 selective inhibitor, ABT-199 on the viability of hESCs, hiPSCs NP and HF. (a) H9 hESCs, FN2.1 hiPSCs, hESCs-derived NP and HF were left untreated or treated with increasing concentrations of ABT-199 (0.1–10 μM) during 15 h (top panel) or treated with CPT(1μM) for 3h or pre-treated with ABT-199 (1μM) for 1 h and then treated with CPT (1μM) during 3h (bottom panel). Cell viability was measured by the XTT/PMS assay 15 h post ABT-199 addition or 6 h after CPT removal. Results are presented as the percentage of the viability of untreated cells. Each bar represents the mean±SEM of three independent experiments performed in quintuplicate. A paired Student's t test was used to compare ABT-199-treated samples with untreated ones (top panel) or CPT plus ABT-199 treated samples to CPT treated ones (bottom panel) *P< 0.05 (b) Representative histograms of PI- stained ABT-199 treated cells over a 20 h period or untreated unfixed cells. Percentage of PI positive cells (late apoptotic or necrotic) was determined by flow cytometric analysis (c) NP were transfected with nt-siRNA or Bcl-2 siRNA and exposed to CPT (1μMduring 3h) 48 h post-transfection. Representative pictures showing nt-siRNA or Bcl-2 siRNA transfected NP treated or not with 1μM CPT for 3 h (48 h post-transfection). Images were captured 12 h after genotoxic removal. The scale bars represent 100 μm. Representative histograms of PI-stained transfected cells 12 h after genotoxic removal (left panel). mRNA expression levels of *bcl-2* in nt-siRNA and Bcl-2siRNA transfected NP were analyzed by Real Time RT-PCR (right panel). GAPDH expression was used as normalizer. Graph shows mRNA fold induction relative to nt-siRNA transfectants arbitrarily set as 1. **P < 0.001.

With the aim to dissect the relative contributions of Bcl-2 and Bcl-xL in pluripotent and progenitor cells fate decisions we used ABT-199, a potent and selective inhibitor of Bcl-2 [40]. To address this issue, we performed viability assays in the presence of increasing concentrations of ABT-199 (0.1μM to 10 μM) during 15 h. We found that inhibition of Bcl-2 neither affects pluripotent cells nor fibroblasts viability (Fig 7a). However, a noticeable loss of cell viability (approximately 40%) was observed in NP after treatment with 10 μM of ABT-199 (Fig 7a). This reduced sensitivity towards ABT-199 was further confirmed by flow cytometry using PI staining after 20h of ABT-199 (1μM) exposure (Fig 7b).

Then, to test the requirement for Bcl-2 in DSBs-induced apoptosis, we pretreated hESCs, hiPSCs, NP and HF with ABT-199 (1μM) and exposed them to CPT. As depicted in Fig 7c no significant changes in the percentage of surviving cells were seen between CPT and CPT plus ABT-199 treated cells. Importantly, we determined the existence of an inverse correlation between Bcl-2 expression and ABT-199 susceptibility.

To further confirm these findings we examined the effects of siRNA-mediated silencing of Bcl-2 expression on NP. Efficient knockdown by Bcl-2 siRNA was verified at 48 h after transfection by real time RT-PCR (Fig 7c). As shown in Fig 7c no appreciable changes were observed in cell viability between Bcl-2 siRNA and nt-siRNA transfectants. Next, we asked whether Bcl-2 downregulation triggers changes in CPT sensitivity. To find the answer, we exposed NP to CPT (1μM) for 3h, 48 h after transfection with 50 nM siRNA, and determined that nt-siRNA and Bcl-2 siRNA transfectants respond equally to the drug (Fig 7c). These results reveal the relatively low importance of Bcl-2 in protecting pluripotent and NP cells from CPT-induced apoptosis.

## Discussion

The study of the detailed mechanisms underlying apoptosis in hESCs and hiPSCs constitute a focus of intense research, because of the promise these pluripotent cells hold for regenerative medicine and the practical need to understand why these cells are so sensitive to external cues. The fact that hESCs and hiPSCs possess the potential to differentiate into all cell types of the three germ layers, expands these interests and raises new prospects for disease modelling as in the case of neurodegenerative diseases. In this regard, another current challenge for researchers is the efficient generation of renewable NP which can further differentiate into neurons, for the study of disease development and cell replacement therapies.

Herein we generated NP from H9 hESCs line and compared their behavior to that of hESCs and hiPSCs after DNA damage triggered by CPT exposure. We determined that both in undifferentiated cells and in hESCs undergoing neural differentiation topoisomerase I inhibition led to ATM activation, p53 nuclear accumulation and its phosphorylation at serine 15 and 46 that culminated in apoptosis. Even though, we observed differences in the kinetics and the extent of apoptosis. In all the cases we found that the apoptotic process encompassed caspase-9 and -3 activation, PARP cleavage and DNA fragmentation.

Furthermore, we found that although NP and HF exhibit similar S-phase populations (S1 Fig) and comparable expression levels of p21^Waf1^ (undetectable in pluripotent stem cells)[26], NP display a higher sensitivity to the S-phase-toxicity caused by CPT than HF. It thus appears that despite the similar cell cycle distribution and the presence of p21^Waf1^ which plays a central role in the coordination of early steps of DNA replication coupled to DSBs repair triggered by CPT [41], additional cell-type specific mechanisms may operate to ultimately dictate cell fate along development.

In response to a death stimulus, a fraction of stress-stabilized p53 rapidly translocates to mitochondria in primary, immortal, and transformed cells [42]. At the mitochondria, p53 interacts with Bcl-xL and Bcl-2 neutralizing their inhibitory effects on pro-apoptotic Bax and Bak. In this sense, Hagn and coworkers proposed that p53 partially fulfils its function at the mitochondria by facilitating the interaction between Bcl-xL and BH3-only proteins, which in turn displaces Bax/Bak from the anti-apoptotic Bcl-2-proteins and consequently promotes mitochondrial outer membrane permeabilization [36]. Herein, we described that abrogation of p53/Bcl-xL and/or p53/Bcl-2 interaction with PFT-μ led to a marked increase in damaged pluripotent stem cells viability and a decrease in spontaneous cell death (Fig 3c) further supporting the concept that even in "primed cell types" anti-apoptotic factors impose thresholds to overcome apoptosis.

Although it remains an open question how the highly primed state is established and maintained in pluripotent stem cells, one potential mechanism could be ascribed to the regulation of the balance between pro- and anti-apoptotic proteins that controls the apoptotic threshold in these cell types. To this end, we analyzed the expression profile of some components of the apoptotic machinery present in hESCs, hiPSCs, NP and HF. We found that pluripotent cells and HF display lower levels of anti-apoptotic Bcl-2 than hESCs undergoing neural differentiation. Additionally, we observed that undifferentiated and immature differentiated progeny show similar expression levels (mRNA levels) of other apoptosis-regulating factors such as *bcl-w*, *bcl-xL*, *mcl-1*, *puma* and *bax*. We also determined that HF exhibit lower levels of *puma* mRNA and higher levels of *blc-w* transcript than the rest of the analyzed cell lines. So, whether this expression profile of apoptotic genes influences the mechanisms underlying HF resistance to CPT or BH3-mimetics remains an open question.

As the height of this threshold is not a fixed property, but rather reflects a dynamic equilibrium between pro- and anti-apoptotic signals, we studied the consequences of perturbing this equilibrium in undifferentiated and differentiated cells. To do so, we used BH3-protein mimetic molecules, ABT-263, WEHI-539 and ABT-199 to investigate: firstly, whether increased occupancy of the BH3-binding pockets is in itself sufficient to initiate a cell death response, and secondly which are the critical events that occur as result of this occupancy which ultimately lead to the onset of cell death. We reasoned that, depending on the expression profile of pro-survival proteins, cell types might differ considerably in their sensitivity to these drugs.

Intuitively, we expected that the abundance of anti-apoptotic Bcl-2 proteins would primarily dictate the cellular sensitivity to BH3 mimetics. In line with our expectations, we found that progenitor cells exhibited a higher sensitivity to ABT-263 than pluripotent cells. Remarkably, by boosting the priming of hESCs, hiPSCs and hESCs-derived NP with ABT-263, we observed that all tested cell types dramatically increased their susceptibility to CPT.

ABT-263 binds to Bcl-2 and Bcl-xL with high affinity and almost 300-fold more weakly to Bcl-w [22]. Thus, the biological effects of this agent appear to be primarily dependent on the inhibition of Bcl-2, Bcl-xL or both of them. Moreover, since ABT-263 has no capacity to interact with Mcl-1 [43], the susceptibility of the tested cell types to this molecule suggests that anti-apoptotic Mcl-1 may not be necessary to guarantee their survival. To this end, the relative dependence of unipotent NP cells on Mcl-1 activity has been previously reported by Crowther and colleagues, who described that mice in which Mcl-1 has been conditionally deleted survive without overt neurologic deficits [44].

To further pinpoint which anti-apoptotic factors are primarily responsible for maintaining survival or death of DNA-damaged pluripotent and progenitor cells we performed anti-Bcl-xL strategies. We found that inhibition of Bcl-xL activity with WEHI-539 or reduction of Bcl-xL expression with siRNA decrease pluripotent and NP viability and dramatically sensitize cells to CPT. These findings highlight the dependency of hESCs, hiPSCs and NP on Bcl-xL for survival.

In further dissecting the contributions of Bcl-2 and Bcl-xL in pluripotent and progenitor cells fate decisions we used the potent and selective Bcl-2 inhibitor, ABT-199 or siRNA-mediated downregulation of Bcl-2. We determined that Bcl-2 inhibition or down-regulation is insufficient to cause apoptosis in pluripotent cells as they resulted almost insensitive to ABT-199 treatment or siRNA-mediated gene silencing. Moreover, impairment of Bcl-2 expression or activity did not affect the extent of the cellular response triggered by CPT in all tested cell types.

The high efficacy of apoptosis induction in hESCs has been shown to depend on multiple mechanisms, on the one hand, the expression of elevated levels of multiple pro-apoptotic proteins [45] and constitutively active Bax sequestered at Golgi rapidly translocating to mitochondria to trigger the cell death program [46]. On the other hand, the unique abbreviated cell cycle of hESCs [47] and the absence of functional G1 and S phase checkpoints operating in these pluripotent cells following DNA damage or replicative stress [48]. Additionally, hESCs are known to have mitochondria that are fragmented, morphologically immature and deficient at oxidative phosphorylation [49, 50]. In this regard, it has been suggested that conditions of mitochondrial fragmentation could facilitate apoptosis [50]. Thus, this unique mitochondrial morphology may somehow predispose hESCs to rapidly undergo apoptosis.

Recently, Crowther and coworkers have demonstrated that primary neural progenitors, like ESC, harbor tonically active Bax at mitochondria, facilitating rapid induction of cell death in response to diverse pro-apoptotic stimuli [44]. Hence, if this molecular setting is also present in hESCs-derived NP, this may explain, at least in part, the increased sensibility displayed by NP to CPT when compared to that of HF.

Despite the fact that pluripotent cells exist in an apoptosis-prone state, their characteristic expression profile of Bcl-2 members may constitute an intrinsic anti-apoptotic “brake” necessary to prevent unwanted cell loss. Importantly, the strengths and weaknesses of this “brake” could be another determinant of their proneness to undergo cell death.

As a whole, in this study we elucidated the consequences of impairing the activities of Bcl-xL and/or Bcl-2 proteins in pluripotent and NP cells after a cell death signal. We provide evidence that Bcl-xL, contrary to Bcl-2, contributes to ensure cell survival and seems to be a primary suppressor of CPT-induced apoptosis in hESCs, hiPSCs and NP. To this end, the more we learn about how stress signals and apoptotic pathways interact in pluripotent and progenitor cells, the better we will be able to predict cellular responses and potentially manipulate these cell types for therapeutic purposes.

## Supporting Information

S1 Fig Cell cycle distribution of asynchronously growing H9 hESCs, FN 2.1 hiPSCs, NP and HF. (TIF)

S2 Fig Morphological changes triggered by ABT-263 treatment and siRNA-mediated downregulation of Bcl-xL. (a) Representative images showing hESCs and hiPSCs colonies grown on Matrigel^™^ coated surfaces, hESCs-derived NP grown onto laminin coated substratum and HF treated or not with 1μM CPT for 3 h, 0.1μM ABT-263 for 15 h or pretreated with 0.1μM ABT-263 for 1h and during CPT exposure (1+3h). (b) Representative pictures showing nt-siRNA or Bcl-xL siRNA transfected hESCs, hiPSCs, NP and HF treated or not with 1μM CPT for 3 h (48h post-transfection). Time points at which images were captured are depicted in the figures. The scale bars represent 100 μm (c) mRNA expression levels of *bcl-xL* in nt-siRNA and Bcl-xL siRNA transfected HF analyzed by Real Time RT-PCR.**P < 0.001.
(TIF)
