# Supplementary figures and images for: Human Pluripotent Stem Cells and Derived Neuroprogenitors Display Differential Degrees of Susceptibility to BH3 Mimetics ABT-263, WEHI-539 and ABT-199
Source: PLoS One. 2016 Mar 31;11(3):e0152607. doi: 10.1371/journal.pone.0152607 (PMC4816327; doi:10.1371/journal.pone.0152607)

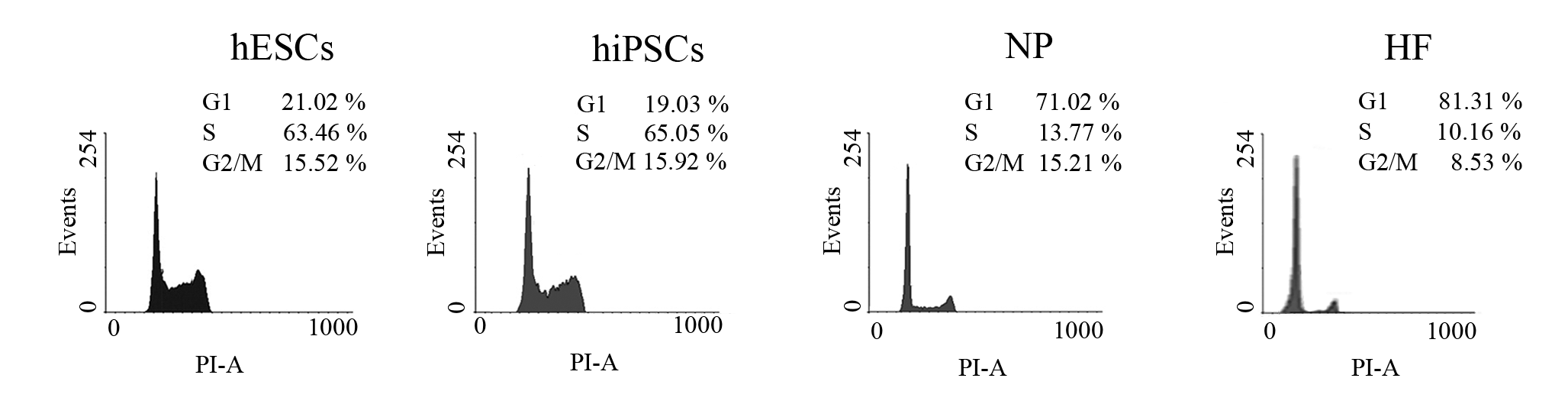

Supplement: S1 Fig — (TIF) [file pone.0152607.s001.tif]

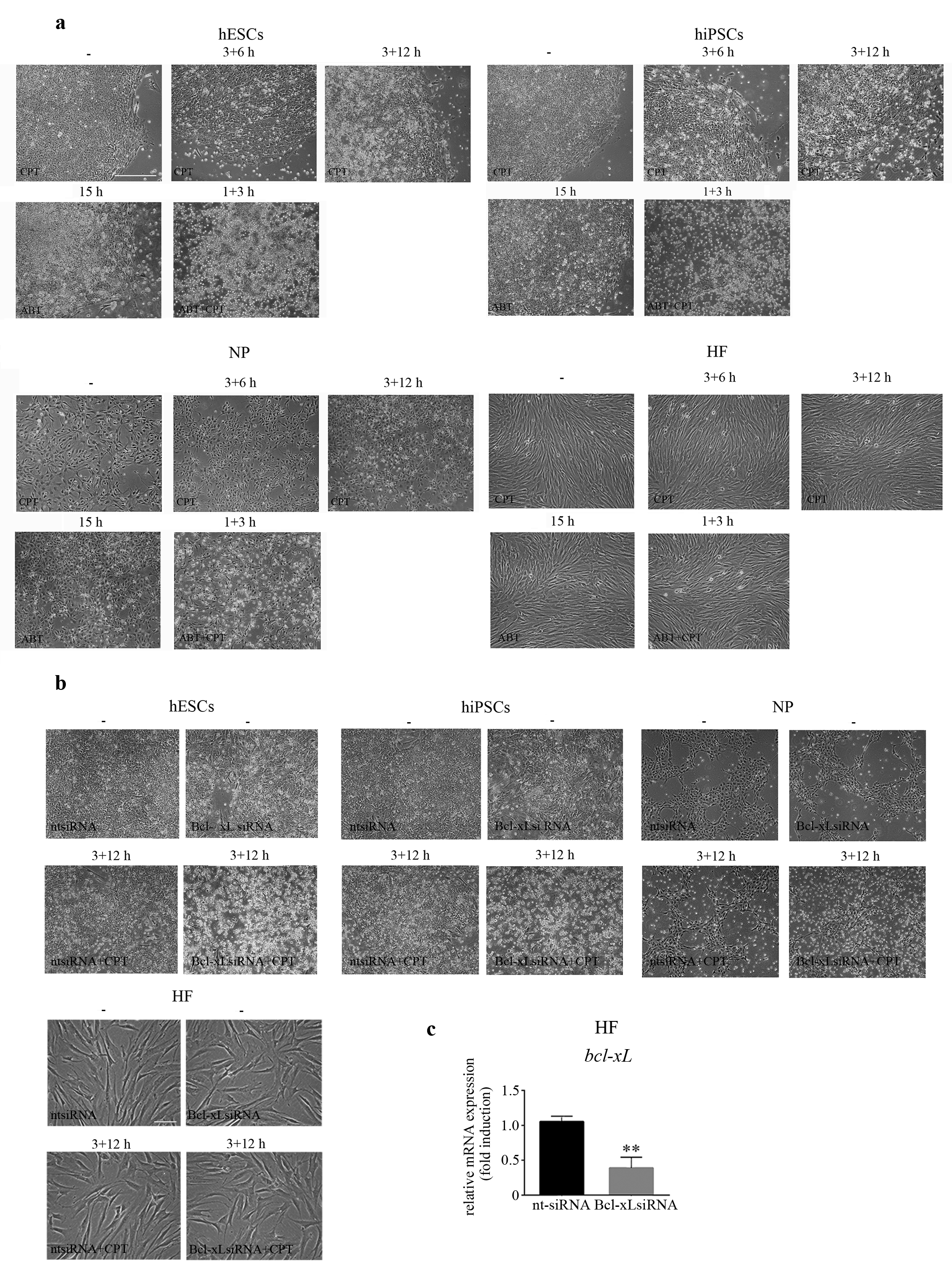

Supplement: S2 Fig — (a) Representative images showing hESCs and hiPSCs colonies grown on Matrigel™ coated surfaces, hESCs-derived NP grown onto laminin coated substratum and HF treated or not with 1μM CPT for 3 h, 0.1μM ABT-263 for 15 h or pretreated with 0.1μM ABT-263 for 1h and during CPT exposure (1+3h). (b) Representative pictures showing nt-siRNA or Bcl-xL siRNA transfected hESCs, hiPSCs, NP and HF treated or not with 1μM CPT for 3 h (48h post-transfection). Time points at which images were captured are depicted in the figures. The scale bars represent 100 μm (c) mRNA expression levels of bcl-xL in nt-siRNA and Bcl-xL siRNA transfected HF analyzed by Real Time RT-PCR.**P < 0.001. (TIF) [file pone.0152607.s002.tif]
